# How electroconvulsive therapy works in the treatment of depression: is it the seizure, the electricity, or both?

**DOI:** 10.1038/s41386-023-01677-2

**Published:** 2023-07-24

**Authors:** Zhi-De Deng, Pei L. Robins, William Regenold, Paul Rohde, Moritz Dannhauer, Sarah H. Lisanby

**Affiliations:** Noninvasive Neuromodulation Unit, Experimental Therapeutics and Pathophysiology Branch, National Institute of Mental Health, Bethesda, MD USA

**Keywords:** Cognitive neuroscience, Medical research

## Abstract

We have known for nearly a century that triggering seizures can treat serious mental illness, but what we do not know is why. Electroconvulsive Therapy (ECT) works faster and better than conventional pharmacological interventions; however, those benefits come with a burden of side effects, most notably memory loss. Disentangling the mechanisms by which ECT exerts rapid therapeutic benefit from the mechanisms driving adverse effects could enable the development of the next generation of seizure therapies that lack the downside of ECT. The latest research suggests that this goal may be attainable because modifications of ECT technique have already yielded improvements in cognitive outcomes without sacrificing efficacy. These modifications involve changes in how the electricity is administered (both where in the brain, and how much), which in turn impacts the characteristics of the resulting seizure. What we do not completely understand is whether it is the changes in the applied electricity, or in the resulting seizure, or both, that are responsible for improved safety. Answering this question may be key to developing the next generation of seizure therapies that lack these adverse side effects, and ushering in novel interventions that are better, faster, and safer than ECT.

## Introduction

Major depressive disorder, a leading cause of disability and death by suicide, is often resistant to available medications and behavioral interventions. Fortunately, electroconvulsive therapy (ECT) offers potent efficacy and rapid onset of action, even after alternative treatments fail. ECT is a medical procedure that entails the induction of a seizure, under anesthesia, for the treatment of severe psychiatric disorders. ECT has an exceptionally broad therapeutic spectrum, with evidence for benefit in unipolar and bipolar disorder (including the acute treatment of depressive and manic episodes, psychotic subtype, as well as relapse prevention), catatonia, schizophrenia, schizoaffective disorder, Parkinson’s disease, epilepsy, status epilepticus, repetitive self-injury in autism, tardive dyskinesia, and neuroleptic malignant syndrome. Despite nearly nine decades of use in psychiatry demonstrating its unparalleled efficacy, the precise mechanisms by which ECT exerts its therapeutic effects and its cognitive side effects remain elusive. Specifically, it is unclear whether it is the electrical stimulus, or the subsequent induced seizure, that is responsible for its therapeutic benefits. This article reviews the latest research that seeks to unpack the mechanisms of ECT and provide insights into whether it is the seizure, the electricity, or both, that underlie its clinical effects. Through a comprehensive review of the literature, we examine the evidence supporting various theories concerning the mechanisms of action of ECT. Earlier work with variations in conventional ECT technique gave clues that modifying the way electricity is applied to the brain (e.g., electrode placement on the head, pulse width, pulse frequency in the train, dosage relative to the minimum required to induce a seizure) could begin to dissociate the efficacy from the side effects of ECT. However, key questions remained unanswered because with conventional ECT, the electrical stimulation and the resulting seizure are always coupled. In other words, you cannot deliver one without the other. This is because conventional ECT delivers very intense and nonfocal stimulation, stimulating nearly the entire brain volume and inducing broadly generalized seizures. Consequently, brain regions associated with both therapeutic efficacy and side effects are all widely stimulated. Because conventional ECT is not optimized to dissociate efficacy from side effects, the field has been in need of novel approaches to unpack the active ingredients of ECT, to allow it to be rebuilt in a manner that is rationally designed and optimized for both safety and efficacy.

This review is timely because we now have available new tools that enable us to unpack ECT and to better disentangle the applied electricity from the resultant seizure. Now, we can employ computational modeling to simulate the electric field (E-field) induced in the brain by ECT, to gain insight into why different forms of ECT have different clinical outcomes, and to better understand differences among individuals who receive the same form of ECT. In addition, we can begin to uncouple the electricity from the seizure by inducing seizures with magnetic stimulation (magnetic seizure therapy, MST) or with weak and more focal E-fields (individualized low amplitude seizure therapy, iLAST). Furthermore, electrical stimulation can be applied without inducing seizure, either at low amplitude as with transcranial magnetic stimulation (TMS) or at high amplitude matching that used with ECT (transcranial electric stimulation therapy, TEST). By contrasting results obtained with these variations on electrical dose and resulting seizure, this article provides a critical evaluation of the available evidence and offers a nuanced perspective on the mechanisms of ECT, with implications for future research and clinical practice.

## Background on ECT

### Definitions and description of the procedure

ECT involves the application of electricity to the brain via electrodes placed on the scalp to induce a generalized tonic–clonic seizure in a person under general anesthesia and muscular paralysis to protect the body from the motor convulsion [1]. The seizure changes numerous aspects of brain chemistry, neural activity, and connectivity, leading to a range of clinical effects [2]. These clinical effects include an exceptionally broad therapeutic spectrum of action (e.g. in depression, psychosis, catatonia, repetitive injurious behaviors in autism, status epilepticus, etc.) as well as a range of cognitive side effects including anterograde and retrograde amnesia [1]. ECT has undergone considerable modifications over the years. Its technical parameters, particularly temporal waveform and electrode placement, play a critical role in determining its efficacy and side effects [3]. The temporal parameters of ECT include pulse shape, pulse width, train frequency, train duration, and train directionality. Modern ECT uses square pulses with pulse widths ranging from 0.25–1 ms. Clinical trials have shown that ultra-brief pulse width of less than 0.5 ms causes significantly fewer adverse effects compared to longer pulse widths but can be equally effective in treating depression when delivered at a sufficient dosage relative to individual seizure threshold [4–10]. Pulses are delivered with alternating polarity with pulse-pair frequency up to 120 Hz, for a maximum train duration of up to 8 s. Pulse amplitude, which governs both the intensity as well as the focality of stimulation, is typically fixed at 800 or 900 mA across patients.

**Fig. 1:**
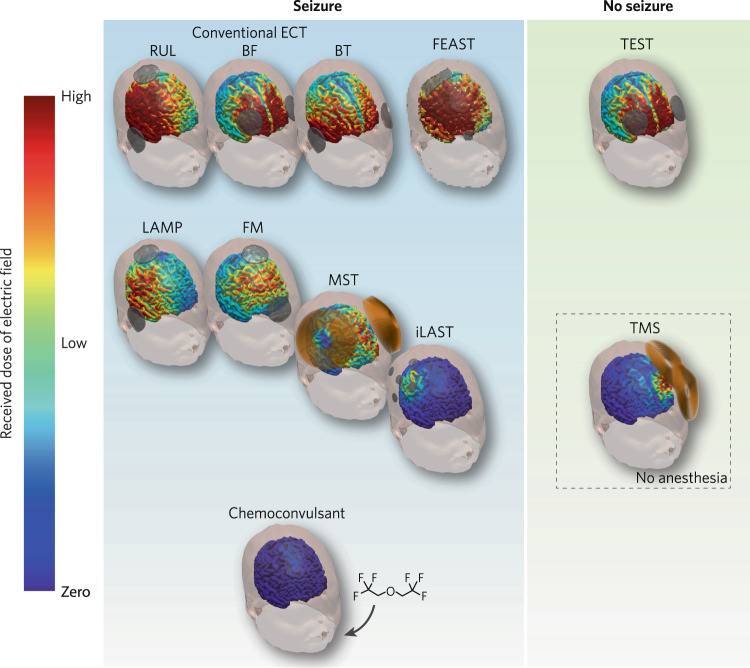
Seizure and nonseizure modalities for treatment of depression. Conventional ECT techniques, with standard right unilateral (RUL), bifrontal (BF), and bitemporal (BT) electrode placements, and experimental technique such as focal electrically administered seizure therapy (FEAST), involve the use of high amplitude E-field for seizure induction. With conventional ECT, the E-field and seizure are always coupled, which does not allow for the study their relative contribution to clinical outcome and cognitive side effects. Techniques that use low amplitude currents for seizure induction include: low amplitude ECT (LAMP), frontomedial (FM) ECT, magnetic seizure therapy (MST), and individualized low amplitude seizure therapy (iLAST). The use of chemoconvulsant for seizure induction involves no use of electricity. These techniques allow us to test the hypothesis that the seizure drives efficacy while the E-field drives side effects. Transcranial electrical stimulation therapy (TEST) is a nonconvulsive modality that use high amplitude E-field. TEST allows us to evaluate the hypothesis that sufficiently high amplitude E-field drive efficacy while the seizure drives side effects. Finally, transcranial magnetic stimulation (TMS) is another nonconvulsive modality that use low amplitude E-field, which allows us to test the hypothesis that low amplitude stimulation, applied repetitively, have a cumulative effect on efficacy with minimal side effects.

The spatial distribution of the induced current flow is a function of electrode shape and size, placement, as well as head anatomy [11]. The current standard of care for ECT commonly uses electrodes with approximately 20-cm^2^ area. Two electrodes are placed in either bitemporal (BT), right unilateral (RUL), or bifrontal (BF) configurations (see Fig. 1). BT ECT, which involves the placement of electrodes 2–3 cm above the midpoint of the line between the outer canthus of the eye and external auditory meatus, is generally considered to be the most efficacious but also with the greatest risk of cognitive side effects [2]. In RUL ECT, one electrode is placed approximately 2.5 cm right of the vertex and the other is positioned in the right BT position. Stimulation by RUL ECT is asymmetrical, with more lateralization to the non-dominant hemisphere compared to BT ECT. In BF ECT, the electrodes are centered 3 cm above the canthus, intended to preferentially target the frontal brain regions. Despite attempts to make the stimulation more focal, when applied at a pulse amplitude of 800 mA, all three conventional electrode placements stimulate most of the brain at or above the threshold for neuronal depolarization [12, 13].

The dosing procedure for ECT often uses a titration scheme to determine the seizure threshold, which is the minimum amount of electrical stimulation required to induce a seizure. In the method of limits procedure, both train frequency and duration are incrementally increased, until seizure activity of adequate duration is observed. The suprathreshold treatments are dosed based on multiples of the seizure threshold charge. In an alternative, so-called “half-age” dosing strategy, the percent energy output of the stimulator is set to half the patient’s age [14]. This rough threshold estimate method was intended to avoid the repeat stimulation in the titration procedure. However, age is only one of many determinants of seizure threshold.

### Brief history of the clinical use of ECT

The earliest form of convulsive therapy to treat psychiatric disorders involved using chemical agents to induce “shock” or seizures. Insulin coma therapy, developed by Viennese psychiatrist Manfred Sakel in the 1930s, and chemoconvulsive therapy, developed by Hungarian psychiatrist Ladislas Meduna around the same time, were two therapy techniques that differ in their approaches. Insulin coma therapy relied manipulating insulin and blood sugar levels to induce a hypoglycemic coma, sometimes with associated convulsions. In contrast, chemoconvulsive therapy focused on using specific chemical agents, such as metrazol, to directly induce seizures [15–18]. The observation that chemically induced seizures can carry powerful therapeutic benefit in the absence of electricity argues that it is the seizure, and not the E-field, that drives the efficacy of ECT—a hypothesis that has not been definitively proven but is under active study using the new tools described below. However, chemoconvulsive therapy was often slow acting, fear inducing, and had life-threatening side effects, leading patients to reject treatment [15]. In the late 1930s, Lucio Bini (1908–1964) and Ugo Cerletti (1877–1963) introduced ECT as a safer and faster-acting alternative to chemoconvulsive therapy [19, 20]. ECT was initially associated with fear, fractures, and memory defects, but the introduction of muscle paralytic agents and general anesthesia in the 1940s and 1950s eliminated some of these issues [21]. The observation that electrically triggered seizures carried more cognitive side effects than chemically induced seizures suggests that the electrical stimulation may drive the cognitive side effects of ECT, though this also remains to be definitively proven. Chemoconvulsive therapy declined as ECT became safer and implemented for various psychiatric disorders including depression and mania, all showing similar efficacy rates [21–23]. Cognitive side effects were still an issue and many of the ECT parameters such as electrode placements, pulse width, waveform, and current intensity were modified to further reduced, though did not eliminate, cognitive side effects [21]. Some of these modifications included changing the waveform from sine wave to brief square pulses, and unilateral electrode placement [15]. When psychoactive drugs were first introduced during the 1950s, they became an easier and safer treatment course with a similar success rate as ECT [15]. This led to a rapid decline in the use of ECT, and ECT was only used when patients were unresponsive to the psychoactive drugs [15]. ECT was further stigmatized as the antipsychiatry movement emerged in the 1970s [24]. During the 1980s and 1990s, researchers began to reevaluate ECT and developed new standards and safety protocols, leading to the establishment of The Practice of Electroconvulsive Therapy: Recommendations for Treatment, Training, and Privileging: a Task Force Report of the American Psychiatric Association as well as multiple placebo controlled studies to evaluate the efficacy of ECT [1, 25]. Since the 1980s, there have been no placebo controlled studies on ECT done, but there have been studies on modifying ECT parameters and applying ECT in conjunction with pharmacotherapies [26].

### Latest evidence for efficacy of ECT

ECT is a highly efficacious treatment for major depression and several other psychiatric disorders. Several meta-analyses support ECT’s superior antidepressant efficacy compared to sham-ECT in randomized controlled trials [25, 27, 28]. Meta-analyses have also suggested a higher overall pooled effect size for ECT compared to repetitive transcranial magnetic stimulation (rTMS) [29–31], and pharmacotherapies [25, 27, 28], including ketamine [32]. Unfortunately, this unparalleled efficacy is often accompanied by cognitive side effects [33], resulting in its underuse. Historically, ECT’s antidepressant effect was thought to be attributed to seizure induction while neurocognitive side effects were associated with electrical stimulation [34–36]. This hypothesis was supported by studies indicating a high efficacy of pharmacologic seizure therapies [37, 38] and lower efficacy of sub-seizure stimulations [39–41]. Complicating this hypothesis, seizures induced by low-dose, right unilateral (RUL) stimulation lacked antidepressant efficacy [42]. Thus, seizure induction may be necessary but insufficient for antidepressant effects [43]. Subsequent studies manipulating electrode placements and stimulus parameters showed that the characteristics and path of the electric current eliciting seizure can have profound effects on antidepressant efficacy and cognitive side effects [4, 44, 45]. RUL ECT’s efficacy when adequately dosed above seizure threshold, inducing seizure in prefrontal regions, supported circuit-specific mediators of ECT efficacy [43]. Neuroimaging studies reiterate region specific contributions [46, 47]. Reductions in frontal network perfusion, metabolism, and connectivity have been associated with antidepressant response [48]. While increases in hippocampal volume have been implicated in both efficacy and cognitive side effects [46, 49, 50], causal mechanisms remain undetermined.

### Latest evidence on safety of ECT

ECT is a safe procedure with risks comparable to surgical procedures requiring general anesthesia [1, 51, 52]. The ECT-related mortality rate was estimated at 2.1 per 100,000 treatments [52]. Studies support its safe administration for patients across the lifespan including pregnant [53], adolescent [54], and geriatric [55] patients. ECT related deaths are rare [51, 56–60]. Patient-matched studies support ECT reduces all-cause mortality rates by decreasing deaths by suicide [56, 61]. Most deaths and adverse medical events from ECT are caused by cardiovascular complications in patients with pre-existing cardiovascular disease [62–65], with less than 1% of patients experiencing respiratory or cardiac events, or prolonged seizure, at some time during their first series of ECT treatments [63]. Patients with underlying cardiac abnormalities can be treated safely with ECT, often with prophylactic administration of beta-blockers to decrease sympathetic drive following seizure [64, 65]. Adverse cognitive events [66, 67] including postictal confusion and memory difficulties [68] can occur. Some cognitive deficits normalize within two-weeks following an ECT course [33]. Persistent cognitive disruptions at 6-months post-course are rare [69, 70]. Some studies report autobiographical memory loss extending for years following treatment, but this remains a contested issue [66, 71, 72]. ECT providers can mitigate adverse cognitive events through using specific stimulation parameters such as RUL electrode placement with an ultra-brief pulse width [4, 8, 73]. Research efforts toward reducing cognitive side effects include pairing ECT with pharmacotherapies [9, 74] and the development of novel seizure therapies as discussed below.

## Theories of mechanism of action

### The amnesia hypothesis

Before there was extensive research aimed at improving adverse cognitive effects, one prominent hypothesis for mechanism of action was that ECT exerted its therapeutic effect by inducing memory loss for the experience of symptoms and for events that may have contributed to the episode of illness [75]. Although it was never evidence-based, this hypothesis unfortunately led to the administration of multiple ECT stimulations within treatment sessions and multiple ECT treatments daily as a means of enhancing ECT-induced amnesia [76]. Despite the lack of evidence, this hypothesis persisted until research showing that ECT techniques that used electrode placements such as right unilateral and bifrontal, or dosing parameters such as ultrabrief pulse widths, induced significantly less amnesia than traditional techniques using bitemporal electrode placement and longer pulse widths, but were equally therapeutic [77, 78]. The observation that reducing overall E-field exposure (by using more focal electrode configurations and more efficient temporal parameters) reduces memory loss without sacrificing efficacy argues for the hypothesis that it is the E-field that drives side effects, while the seizure drives efficacy.

### The anticonvulsant hypothesis

The anticonvulsant hypothesis contends that ECT’s antidepressant effect is mediated by a postictal inhibitory surge in regions of the prefrontal cortex (PFC), which both terminates the immediate seizure and enhances GABAergic function [79]. This theory is derived from the GABA deficit model of affective disorders [80]. It was supported by evidence showing ECT increases seizure threshold [81, 82] and preclinical models using electroconvulsive shock, indicating seizure threshold increases were GABA selective [83, 84]. Later studies showed that increases in seizure threshold was correlated with antidepressant response [45, 85], specifically, ECT responders experienced greater threshold increases than non-responders [86]. PFC specificity was inferred from findings that high-dose RUL and BT ECT, both initiating seizures in PFC regions, produced the same magnitude of seizure threshold increase and antidepressant response. Low-dose RUL ECT, initiating seizures in motor cortex, lacked antidepressant efficacy and resulted in minimal changes in seizure threshold [86]. Other electrophysiological measures using TMS evoked EEG potential and long-interval cortical inhibition, which are indices of GABAergic interneuron function, have been shown to predict suicidal ideation outcomes following seizure therapy [87]. If it is the inhibitory response to the seizure that drives efficacy, that poses the intriguing possibility that inducing that inhibitory response in the absence of seizure might be a means of achieving efficacy without needing a seizure, or electricity for that matter. However, subsequent investigations have not replicated key findings of the anticonvulsant hypothesis. Studies have failed to reproduce the correlation between seizure threshold increases and antidepressant response [88–90]. Magnetic resonance spectroscopy (MRS) studies of GABA concentration changes during an ECT course report either no significant change [91] or GABA increases without a relationship to antidepressant outcome [92]. Further, in a study of ECT’s effect on plasma concentrations of neuroactive steroids that are known to modulate GABA receptor function, no change was found before and after ECT treatment [93]. In addition to these failed replications, the anticonvulsant hypothesis has conceptual limitations. It does not provide a rationale for why an inhibitory cascade is necessary for antidepressant response or account for ECT’s efficacy in treating psychiatric disorders other than major depression [94, 95]. Abbott and colleagues argue for integrating the anticonvulsant and neurogenesis hypotheses [96]. They contend that a frontal inhibitory surge and subsequent reductions in metabolism and perfusion are key to an acute response, but neurotrophic changes in the medial temporal lobes are required for sustained remission [96].

### The neurogenesis hypothesis: structural neuroplasticity and cellular proliferation

The neurogenesis hypothesis posits that the therapeutic effects of ECT depend on increasing the number of neurons or the connections among neurons. The hypothesis was originally tested in experiments using an animal model of ECT that showed neurotrophic effects on rodent hippocampus from either a single or a series of electroconvulsive seizures, that included increases in the following: synaptic proteins suggesting synaptogenesis [97, 98]; granule cell and mossy fiber sprouting [99, 100]; newborn cells in the dentate gyrus [101], and bromodeoxyuridine (BrdU)-positive cells in the dentate gyrus [102, 103]. Further studies showed that electroconvulsive seizures amplified the signaling of growth factors including brain derived growth factor (BDNF) in numerous brain areas and vascular endothelial growth factor (VEGF) in the hippocampus [104, 105]. Cell proliferative effects of electroconvulsive seizures were also observed in rat frontal cortex; however, newly divided cells differentiated into endothelial cells or oligodendrocytes, but not neurons [106]. Extended to an adult nonhuman primate model, electroconvulsive seizures increased precursor cell proliferation in the subgranular zone of the hippocampal dentate gyrus in the monkey hippocampus, with most precursor cells differentiating into neurons or endothelial cells [107]. Interestingly, epileptic seizures also induce similar neurogenesis effects in the absence of electrical stimulation, so if neurogenesis drives efficacy, this will argue for the seizure, and not the E-field, driving efficacy.

The neurogenesis hypothesis was eventually tested indirectly in patients largely through studies measuring BDNF blood levels and or by neuroimaging brain structure, function, and metabolism pre- and post-ECT. Two recent meta-analyses of BDNF blood levels studies concluded that BDNF increases after ECT treatment, but there is considerable heterogeneity among blood levels and no clear relationship between change in BDNF and change in depressive symptoms [108, 109]. Neuroimaging studies have been reviewed recently [47, 110] and will be discussed in detail below under the role of anatomical and functional neuroplastic changes in the efficacy and adverse effects of ECT.

### Role of the electric field

The induced E-field that is delivered to different brain regions by ECT can be quantified to assess its relation to other measures (i.e., neuroplastic and clinical outcomes). The E-field can be estimated using models of the head roughly approximated by spherical shells [12] or using realistic models derived from individual MRI [13]. With these computational head models, the impact of ECT parameters, such as electrode configuration and pulse amplitude, can be simulated for individual ECT procedures, to help explain changes in brain volume and clinical outcomes. These computational modeling studies demonstrate that conventional ECT electrode sizes and placements, when used at the convectional current amplitudes of 800–900 mA, stimulate nearly the entire brain at intensities that are far in excess of the threshold for neuronal depolarization. These simulations are consistent with in vivo studies in nonhuman primates [111]. The three commonly used electrode placements (RUL, BT, and BF) show different seizure thresholds believed to be related to different E-field exposure (see Fig. 1), as well as regionally specific differences in seizure susceptibility. However, while there are differences in the spatial distribution across these three electrode placements, all three are fundamentally non-focal in the sense that they stimulate almost 100% of the brain above the threshold for neuronal depolarization [12, 13]. In addition, there is marked variability across individuals in the induced E-field even for a fixed electrode placement. This is due to the high sensitivity of the ECT E-field to the impedances of head tissues, especially to the thickness and electrical conductivity of the skull [112].

The relationships between the ECT induced E-field, seizure characteristics, physiological changes, clinical outcome, and cognitive side effects are under investigation. ECT has been shown to produce volume increases in a wide array of cortical and subcortical regions [113]. The widespread brain volume expansion evident in bilateral hemisphere, even when unilateral electrode placement is used [114]. There is a reproducible relationship between induced E-field strength at the medial temporal regions, particularly the hippocampus and the change in the regional gray matter volume [50, 114, 115]. In other brain regions, the volume increase is not correlated with the induced E-field strength, suggesting that the physiological effect of the electrical stimulation is more localized than the field map would suggest, and that the extensive brain volume increase is related to the cumulative effect of the seizure [114]. The relationships between the hippocampal E-field and clinical outcome, and between hippocampal volume change and clinical outcome, is complex. In a recent randomized clinical trial comparing RUL ECT with different pulse amplitudes (600, 700, and 800 mA), structural equation modeling of the relationship between hippocampal E-field, hippocampal volume change, and depression score was performed [50]. It was found that the hippocampal E-field was not directly related to antidepressant outcomes. However, the hippocampal E-field has a positive linear relationship with hippocampal volume change; this volume change was in turn negatively correlated with the change in the depression score. That is, the hippocampal volume mediated the relationship between E-field and antidepressant response, but in a way that suppresses E-field’s direct effect. With the same modeling method, it was shown that the hippocampal E-field had a direct effect on cognitive impairment as measured by phonemic fluency, that was not mediated by volume change [50]. This suggests that inducing seizures with no hippocampal E-field exposure (as can be done with MST and iLAST described below) could disentangle the impact of the E-field from the neuroplastic effects of the seizures.

Computational modeling of E-field is an active area of research and development and requires further validation. E-field modeling with human head models has undergone validation for transcranial electrical stimulation using in vivo intracranial recordings [116, 117] and magnetic resonance current density reconstruction approaches [118]. Additionally, ECT stimulus modeling for amplitude-determined seizure titration has been validated with non-human primates [119, 120]. To enhance the accuracy of E-field modeling, further improvements can be made in tissue segmentations and conductivity values.

### Role of seizure expression

EEG monitoring is commonly used during ECT to assess the quality and duration of the induced seizures and to ensure patient safety. Peri-treatment EEG can be divided into several phases: preictal (before stimulus and seizure), recruitment (high frequency activity, EEG gains in amplitude), polyspike (a mixture of frequencies reflecting a balance of excitatory and inhibitory processes), slowing (reduced amplitude and frequency as inhibitory mechanisms dominate), and postictal (bioelectric suppression) phases [121, 122]. ECT treatment responses are often associated with long duration and greater amplitude of seizures [123, 124]. However, recent studies have found that seizure duration may not be a reliable predictor of treatment outcome and that it is more complex [123, 125]. Meta-analyses have shown that certain ictal EEG indices, including postictal suppression, early and mid-ictal amplitude, recruitment phase duration, symmetry and interhemispheric coherence, spectral power, seizure quality or strength, global EEG power, and Largest Lyapunov Exponent, are associated with superior clinical outcomes [124, 126]. Among these indices, postictal suppression appears to be commonly associated with improved clinical outcomes and as a predictor for treatment outcomes [124]. One study found that postictal suppression during the first ECT treatment predicts long-term clinical response, defined as after 12 ECT sessions, with a response rate of 74% for patients who exhibit postictal suppression during their first ECT session compared to 55% for patients who did not [127]. Additionally, recent studies suggest that central-positive complexes may link to better clinical outcomes, which may provide new insights into the mechanisms reflecting generalized seizure activity [128, 129]. However, both ECT treatment techniques and anesthesia influence ictal EEG and therapeutic outcomes [124, 130]. During general anesthesia, an increase in the bispectral index at seizure induction has been correlated with high seizure quality [130]. ECT treatment techniques such as electrode placement, pulse width, waveform, and dose relative to seizure threshold can affect the seizure duration and intensity of certain seizure phases [131].

The seizure quality induced by ECT has been associated with the severity and occurrence of cognitive side effects or post-ictal confusion [132]. One study found that a larger increase in global delta power and theta activity in the left frontotemporal regions correlated with longer recovery time and an increased magnitude of retrograde amnesia for autobiographical memory [132]. Similarly, another studies found that an increase in ictal theta power was correlated with cognitive impairment [133]. Another study suggests that longer clinical reorientation time may be associated with slower postictal EEG recovery [134]. However, other studies have shown no association between peri-ictal EEG and short-term cognitive side effects [121]. Similar to anesthesia influencing treatment outcome, anesthesia can also contribute to cognitive impairment. For instance, a longer period of memory impairment was associated with lower bispectral index values [135]. The role of seizure expression and the efficacy and cognitive effects of ECT is complex.

The relationship between the E-field, seizure characteristics, and clinical outcomes is not well understood. A pilot study identified the whole brain E-field is significantly correlated with ictal theta power, which is in turn correlated with phonemic and semantic fluency after ECT [133, 136]. With conventional ECT, it is impossible to completely disentangle whether it is the treatment technique (which drives the spatial and temporal aspects of the delivered E-field) or the differences in seizure expression that drives the observed clinical differences, since E-field and seizure expression are so tightly coupled.

### Role of anatomical and functional neuroplastic changes in ECT efficacy and adverse effects

There is a growing literature on the neuroplastic effects observed in the brains of patients receiving ECT. We will focus here on evidence from structural and functional magnetic resonance imaging, which have been the most prominent methods. Parallel to the animal model literature, much of the research on neuroplastic changes with ECT has investigated hippocampal volume changes and whether these changes correlate with either antidepressant response or adverse cognitive effects. Nordanskog et al. were the first to show an increase in hippocampal volume with ECT [137]. In 12 patients receiving ECT for depression, they found that ECT, using predominantly RUL electrode placement, significantly increased hippocampal volume bilaterally when measured within one week before and after a course of ECT averaging 10 treatments. In a follow up study, these patients had MRIs at 6-month post-ECT course which revealed that hippocampal volumes had decreased back to pre-ECT levels and that change in hippocampal volume was unrelated to antidepressant or cognitive outcomes. Abbott et al. reported increased volume in right (but not left) hippocampal cornu ammonis (CA2/3), dentate gyrus and subiculum regions in 19 depressed patients following a course of RUL ECT, which did not correlate with change in depressive symptoms or cognition. However, they also found that right hippocampal connectivity increased in the right temporal lobe, and this correlated with decreasing depressive symptoms [46]. Jorgenson et al. reported hippocampal and amygdala volumes increased compared to baseline at 1 and 4 weeks after a course of largely bitemporal ECT in 19 patients with severe unipolar or bipolar depression [138]. These volume changes were accompanied by diffusion tensor imaging (DTI)-measured reduced anisotropy and diffusivity of the hippocampus at one week after the ECT course, but no changes in brain metabolites measured by proton magnetic resonance spectroscopy nor in blood BDNF level. None of the imaging measures correlated with change in depressive symptoms or with blood BDNF level. In a larger study of 66 depressed patients receiving predominantly RUL ECT, Bouckaert et al. found that hippocampal volume increased bilaterally from baseline to one week post-ECT course, but not at 6 months post-ECT course [139]. They also measured serum levels of BDNF, which did not change with ECT. There were no significant correlations among changes in hippocampal volume, depressive symptoms or BDNF serum levels. Another long-term follow up study also found that initial increases in gray matter volumes in right hippocampus and both thalami over an ECT course were lost 10–36 months later in 16 individuals with major depressive disorder [140]. Sartorius et al. reported post-ECT course gray matter volume increases in hippocampus and amygdala that were more robust in the right hemisphere, but did not correlate with clinical variables including change in depressive symptoms or cognitive performance in 92 patients treated predominantly with RUL ECT for depression [141]. Camilleri et al. reported gray matter volume increases in the right hippocampus/amygdala from a baseline scan within a week of ECT to a 2–16 day post-ECT course scan in 85 patients who received ECT for depression compared to 86 healthy controls; however, they did not comment on the relationship of their volume change finding to clinical outcomes [142]. Two studies have shown that increases in hippocampal volume with ECT have correlated with adverse cognitive effects. Oostrom et al. reported that increases in hippocampal volume over a course of ECT correlated with decreased performance on cognitive outcome measures [143]. Laroy et al. scanned and assessed the cognition of 66 adults aged 55 years and older 6 months post-ECT course for depression and found that a greater increase in right hippocampal predicted less improvement on a measure of visual memory [144]. The hypothesis that increases in hippocampal volume are a function of an ECT-related neurotrophic effect was suggested by the finding that a regulatory single nucleotide polymorphism (SNP) in the VEGF (but not the BDNF) gene associates with greater hippocampal volume increases following a course of RUL ECT in 61 older adults (55 years and older) with major depressive disorder [145].

A study of hippocampal function pre- and post-ECT using arterial spin-labeled functional magnetic resonance imaging showed that regional cerebral blood flow (CBF), indicative of hippocampal function, increased in the right anterior hippocampus in all 57 patients receiving ECT for depression following both the second treatment and after a full course of treatment. However, increases in hippocampal CBF post-ECT course were greater in nonresponders than in responders [146]. To summarize studies to date, ECT-related increases in hippocampal volume and function may represent ECT-induced neuroplasticity but seem to be transient and have no clear relationship to ECT antidepressant efficacy and adverse cognitive effects.

There is a smaller body of literature concerning ECT-induced neuroplasticity in brain regions other than the hippocampus and in white matter. Pimia et al. reported increased thickness in the bilateral anterior cingulate cortex (ACC), inferior and superior temporal, parahippocampal, entorhinal and fusiform cortex and in distributed prefrontal areas over a course of largely RUL ECT in 29 patients with unipolar or bipolar depression [147]. Increasing cortical thickness of fusiform, superior, and inferior temporal gyri correlated with decreasing depressive symptoms. Interestingly, a relationship between increase in ACC thickness and symptom improvement was observed only in responders. Lyden et al., used DTI to assess potentially neuroplastic changes in 20 patients receiving a course of RUL or BT ECT for a major depressive episode, found increases in fractional anisotropy, suggesting increased white matter microstructural integrity, in bilateral anterior cingulum, forceps minor, and left superior longitudinal fasciculus (SLF) [148]. These fractional anisotropy increases correlated with decreases in depressive symptoms, suggesting that ECT modulates depression-related altered white matter microstructure in mood-regulating dorsal fronto–limbic pathways.

Several studies have investigated the effects of ECT on functional connectivity at the network level, yielding insightful findings. Whole-brain analysis revealed significant effects of ECT on functional connectivity in various networks, including the default mode network (DMN), central executive network (CEN), sensorimotor network, and cerebellar posterior lobe [149]. ECT was observed to increase connectivity within the DMN and between the DMN and CEN. These results align with previous reports indicating that ECT can normalize the connectivity between the DMN and CEN [150]. Correlations can be observed between specific patterns of connectivity changes and the improvement of depressive symptoms following ECT. Notably, alterations in functional connectivity between the medial prefrontal cortex and ventrolateral prefrontal cortex [149], as well as connectivity between the dorsomedial prefrontal cortex and posterior cingulate cortex [151], have been linked to therapeutic outcomes. A summary of the neuroplastic effects of ECT is provided in Table 1.

**Table 1:** Overview of key neuroplastic effects observed in patients receiving ECT.

Brain region	Neuroplastic effect	Correlation with outcome
Hippocampus	Increased volume	No clear relationship with antidepressant response or cognitive effects
	Reduced anisotropy and diffusivity	
	Increased functional connectivity to right temporal lobe, angular gyrus	Correlated with depressive symptom improvement
Anterior cingulate cortex	Increased thickness	Correlated with depressive symptom improvement (only in responders)
Temporal cortex	Increased volume	
White matter	Increased fractional anisotropy in specific pathways	Correlated with depressive symptom improvement
Dorsolateral prefrontal cortex	Decreased global functional connectivity	Correlated with depressive symptom improvement
Dorsomedial prefrontal cortex	Increased functional connectivity to posterior cingulate cortex	Correlated with depressive symptom improvement
Default mode network	Increased functional connectivity within	Baseline connectivity related to depressive symptom improvement
	Increased functional connectivity to central executive network	

A model of the neurobiological effects of ECT has been proposed [110]. The brain of individuals with depression brain prior to treatment is characterized by low plastic potential, resulting in high symptom load. Each ECT session, which involves electrical stimulation and seizure induction, temporarily disrupts brain function, including potentially postictal confusion. This disruption results in physiological changes, such as reduced N-acetylaspartate, functional connectivity, and white matter integrity. In response to these disruptive effects, the brain exhibits a temporary enhancement of neuroplasticity, leading to an increase in brain tissue volume and enhanced connectivity. The combination of disruption and neuroplastic effects ultimately leads to a rewiring of neural circuits associated with depressive symptoms. The model posits that in the case of excessive E-field dosing, there is more widespread volume effects and disruptive effects; antidepressant response is accompanied by cognitive impairment. In the case of insufficient dosing, there is minimal antidepressant response and no cognitive side effects.

## New tools to uncouple the E-field from the seizure, and what they can teach us about the mechanisms of action of ECT

### Seizure induction with low amplitude electrical current

Seizure induction with low amplitude E-fields is a relatively new approach to seizure therapy that aims to separate the therapeutic benefits of seizure induction from the side effects associated with the induced E-field. The hypothesis behind this approach is that the therapeutic efficacy of ECT is primarily driven by the induced seizure, while the adverse side effects are driven by the E-field that is generated by the stimulus. There are several approaches to inducing seizures with lower stimulation dose, including low amplitude ECT (LAMP), individualized low amplitude seizure therapy (iLAST), and magnetic seizure therapy (MST).

#### Low amplitude ECT (LAMP)

Even though stimulus pulse amplitude is a major driver of the E-field intensity and focality in the brain, it has received surprisingly little attention in dosage optimization. As mentioned previously, at conventional pulse amplitudes of 800 or 900 mA, ECT induces an E-field strength that substantially exceeds the neural activation threshold in most brain structures [12, 13], resulting in widespread, nonfocal stimulation. The induced field strength is much higher than necessary to induce seizures. At these pulse amplitudes, more than 90% of the brain is exposed to suprathreshold stimulation. While this nonfocal stimulation may engage the broad neural system that contribute to antidepressant efficacy, the non-targeted brain regions that contributes to the cognitive side effects are also stimulated intensely. A simple solution is reduction of the pulse amplitude [11]. Since the E-field is directly proportional to the pulse amplitude, this amplitude reduction linearly decreases the stimulation intensity. Focality of the stimulation is also reduced approximately linearly over a range of current amplitudes, though the sensitivity depends on interelectrode distance [11]. ECT studies dating back to the 1940s as well as several small recent studies demonstrated the feasibility of low-amplitude currents inducing seizures [152–155]. In a pilot study of LAMP in 22 patients [156], ultrabrief pulse RUL electrode placement was used; seizure threshold was titrated using the current amplitude of 500 mA, starting with frequency of 20 Hz and titrated up if seizures were not induced. Subsequent treatments were delivered at 6 times seizure threshold, with current amplitude fixed at 500 mA. All 22 patients seized in the first session, had a quick time to reorientation, and treatment was efficacious for both depressive and psychotic symptoms.

#### Individualized low amplitude seizure therapy (iLAST), focal electrically administered seizure therapy (FEAST), and frontomedial ECT

Another drawback of conventional ECT is that the pulse amplitude is fixed for all patients. Even when seizure threshold is titrated and the dose is set relative to seizure threshold, this is done by increasing the number of pulses, via increases in train duration and/or frequency, but not the pulse amplitude. which does not compensate for the individual variation of the E-field strength in the brain [11]. Consequently, individual variations in anatomy, including tissue thicknesses and electrical properties, result in variable strength and focality of the induced E-field, which could contribute to differences in side effects and therapeutic efficacy across patients [112]. Therefore, individualization of the pulse amplitude in ECT could help maintain a consistent E-field exposure of the brain to compensate for individual anatomical differences. The advantage of amplitude individualization has been demonstrated in a nonhuman primate study [119]. Four rhesus macaques each received ECT with four electrode configurations: bitemporal, bifrontal, right unilateral, and frontomedial. Seizure threshold was determined by an ascending method-of-limits titration of the pulse amplitude [119]. Induced E-field was simulated in each subject using the finite element methods and the simulation results showed that the variation in stimulated brain volume with individualized low current amplitude is reduced compared to fixed high current amplitude. This suggested that amplitude individualization could be a means of compensating for interindividual variability in anatomy and neurophysiological excitability. In a proposed human variation of the iLAST technique, the amplitude titration method is combined with the use of a multielectrode stimulation configuration to further optimize the focality (NCT03895658).

Among the approaches that make ECT more focal, electrode placement has been the subject of the most intensive investigation. The shift from BT to RUL and BF electrode placements is representative of the move toward more focal electrical stimulus delivery, based on the assumption that by reducing the spacing between the electrodes and placing them over the desirable brain regions, the direct stimulation and seizure intensity can be reduced, thereby reducing side effects. ECT focality can be further refined by manipulation of the electrode size, shape, spacing, and current amplitude [11]. For example, focal electrically administered seizure therapy (FEAST; [157]) uses a smaller anodal electrode and a single or multiple (bigger) cathodal electrodes showing more lateralized seizures at lower thresholds. Another example is the frontomedial (FM) electrode placement, in which one electrode is placed on midline prefrontal cortex and the other on the vertex, and uses low current to further stimulation focality [155]. While FEAST and FM reduce the percent of total brain volume exposed to suprathreshold E-fields, they still stimulate more than half the brain, and importantly, they both stimulate the hippocampus at intensities strong enough to induce neural depolarization [13]. Further improvements to focality are possible with a use of multielectrodes, for example, using the 4-by-1 montage used in high-definition transcranial direct current stimulation (HD-tDCS [158]). The 4-by-1 electrode array can be placed to target different brain regions, for example, midline prefrontal cortex or motor cortex. In addition, the inter-electrode distance (“radius”) within the array can be adjusted to create different depth–focality profiles. These features allow testing of different hypotheses related to the importance of seizure initiation site and stimulation focality (NCT03895658).

#### Magnetic seizure therapy (MST): testing the hypothesis that the seizure conveys efficacy and sparing E-field exposure to hippocampus protects memory

MST is an emerging alternative for inducing seizures that presents significant advantages over conventional ECT techniques. One of the most significant benefits is that magnetic stimulation generates E-fields that are mainly tangential to the head surface, rendering them less sensitive to radial variations in electrical conductivity between various tissue compartments. This overcomes a major limitation in ECT, which is that due to the low conductivity of skull, a large proportion of the ECT current gets shunted by the scalp. The amount of ECT current entering the brain is highly sensitive to skull thickness and electrical conductivity. MST significantly reduces the inter-individual variability of the induced E-fields compared to ECT [112], which may lead to more consistent clinical outcome. Moreover, MST produces more superficial E-field, potentially avoiding direct stimulation of deeper temporal regions [12, 159]. Furthermore, MST uses a less efficient sinusoidal pulse waveform, further reducing its stimulation strength. Computational studies suggest that the MST-induced E-field relative to threshold is 3–6 times weaker than ECT, and it activates only 20% of the brain, in contrast to ECT, which activates over 90% of the brain [12, 13]. Nonhuman primate studies confirm that MST induces more focal E-fields and seizures that spare the temporal lobe [160]. Moreover, there is evidence in nonhuman primates that MST has significantly less cognitive side effects than ECT and is comparable to the effects of anesthesia alone [161–163]. Clinical studies to date have demonstrated that MST has similar antidepressant efficacy compared to ECT; and MST showed less cognitive impairment on several measures, including immediate and delayed recall of words, visual–spatial immediate and delayed memory, and verbal fluency (for review and meta-analysis, see [164]). An adequately powered confirmatory efficacy study is currently underway to provide definitive evidence of whether MST is noninferior to ECT in terms of efficacy, while retaining its relatively benign cognitive side effect profile [165].

### Low amplitude electrical currents without seizure induction: testing the hypothesis that low amplitude currents, applied a sufficient number of times, have a cumulative effect on efficacy

Transcranial magnetic stimulation (TMS) is a non-invasive neuromodulation technique that utilizes magnetic fields to induce electrical currents in the brain (see Fig. 1). TMS has FDA approval for the treatment of depression in adults who have not responded adequately to antidepressant medications. The FDA has also approved TMS for the treatment of obsessive-compulsive disorder, migraine, smoking cessation, and anxious depression [166]. TMS has been shown to have both excitatory and inhibitory effects on cortical activity, depending on the parameters of stimulation, such as coil configuration, current direction, pulse waveform, temporal pattern (fixed frequency repetitive or mixed frequency bursting patterns), intensity, and total number of pulses. In terms of shaping the induced TMS E-field, coils with different geometry, size, and orientation can be used to target specific brain regions. Regardless of coil design, the stimulation depth of magnetic is limited compared to electrical stimulation [12]. In terms of temporal pulse patterns, it has become a heuristic that when TMS is delivered at a high frequency (>5 Hz) or an intermittent theta burst stimulation (iTBS) pattern, it can result in facilitation of synaptic transmission, leading to an long-term potentiation (LTP)-like increase in excitatory neural activity [167]. This is the rationale for the use of high frequency rTMS and iTBS on the left DLPFC to correct the region’s hypoactivity in depression. Conversely, when TMS is delivered at a low frequency (1 Hz) or a continuous theta burst stimulation (cTBS) pattern, it can result in long-term depression (LTD)-like suppression of neural activity [168]. Sequential bilateral stimulation, a technique that combines high frequency rTMS over the left DLFPC and low frequency rTMS over the right DLPFC, has been developed to rebalance the activity between the two hemisphere and potentially enhance the antidepressant effect [169]. These heuristics were derived from observations of group effects, it is important to note that individuals exhibit significant variations, and that changing other temporal parameters such as the number of pulses during a session can potentially reverse the inhibition or excitation effects [170].

Another temporal parameter under investigation is the frequency of treatment sessions. The idea of densely packing many pulses into a short duration, via longer burst sequences per session and/or accelerated treatment schedules, assumes that antidepressant treatment effect builds cumulatively with repeated stimulation and that accelerated stimulation can lead to accelerated response. By increasing the total number of sessions delivered within a shorter timeframe, it is hypothesized that accelerated TMS may have comparable or even superior outcomes to traditional TMS protocols [171]. One study compared accelerated high-frequency rTMS and iTBS delivered over the left DLPFC under MRI guidance, at 5 sessions per day for 4 days, do not differ in clinical efficacy [172]. The overall response and remission rates of these accelerated protocols were similar to standard daily rTMS paradigms, leading to the conjecture that within current stimulation guidelines, the antidepressant effect of TMS has reached a ceiling effect. However, in the recently approved SNT protocol, in which high-dose iTBS was delivered under individualized functional connectivity guidance, at 10 sessions per day for 5 days, showed unprecedented remission rate of ~90% [173]. It is yet to be shown which aspect of the SNT protocol contributed the most to its remarkable antidepressant efficacy.

### High amplitude electrical currents without seizure induction: testing the hypothesis that sufficiently high amplitude electric pulses drive efficacy while the seizure drives side effects

Transcranial electric stimulation therapy (TEST), previously called nonconvulsive electrotherapy, involves BF electric brain stimulation at a dose below seizure threshold applied exactly as standard ECT. This procedure is performed under general anesthesia similar to conventional ECT, and with a FDA-cleared ECT device that can deliver a range of subseizure threshold stimulation doses [174]. Since TEST, like standard ECT, delivers pulses at a high current amplitude, it induces a brain E-field distribution like that of BF ECT (see Fig. 1). However, since the frequency and duration of the stimulus are very low compared with ECT, the stimulus lacks the energy to induce a generalized seizure. Therefore, rather than aiming to decrease adverse cognitive effects through lower amplitude and/or more focal stimulation, TEST intends to limit adverse cognitive effects by eliminating the seizure. This approach tests the hypothesis that sufficiently high amplitude electric pulses drive ECT efficacy while the seizure drives ECT side effects. This challenges the long-standing theory of ECT therapeutic mechanism, which holds that the generalized seizure is necessary for the therapeutic effect of ECT. With a significantly lower dose stimulus than ECT and lacking generalized seizure induction, the rationale for the possible therapeutic effect is based on its BF stimulation and the robust evidence implicating frontal lobe regions and frontolimbic neural circuits in depression and in antidepressant treatment response [175–179]. This evidence also supports the choice of stimulation site for the only other FDA-approved brain stimulation therapy for treatment-resistant depression (TRD)—rTMS. Randomized clinical trials showing that low-dose RUL ECT, in contrast to bilateral forms of ECT, can elicit a generalized seizure without being therapeutic, indicate that regional distribution of the stimulus within the brain is critical to antidepressant effect [42, 77]. The potential therapeutic mechanism of action of TEST is unclear; however, the potential for a neurotrophic effect without inducing seizures is suggested by a study finding that 10 min of daily (for 10 days) subconvulsive electrical stimulation using a deep brain stimulator induced increases in dorsal hippocampus BDNF levels that are comparable to those induced by daily ECS in a rat chronic mild stress model of depression [180]. To date, evidence for the safety and efficacy of TEST as an antidepressant treatment is limited to an open-label clinical trial with 11 completing participants with TRD who had thrice-weekly treatments [174]. In this study, there were no serious adverse events, and adverse cognitive effects were less than typically observed for standard BF ECT. TRD response and remission rates (73% and 55%, respectively) were comparable to those typically observed in ECT trials. Sham or ECT-controlled trials are needed to determine the safety and antidepressant efficacy of TEST.

## Summary and synthesis

Here we summarize what the available evidence has to say about the tantalizing question of whether it is the seizure or the E-field, or both, which drive the efficacy and side effects of ECT in the treatment of depression. Studies using conventional ECT cannot provide a satisfying answer to this question, because the E-field and the seizure are always coupled. Conventional ECT induces strong and diffuse E-fields coupled with broadly generalized seizures, which carry significant therapeutic benefits as well as substantial risk of cognitive side effects. While modifying spatial and temporal parameters (though electrode placement and pulse width, respectively) have lowered cognitive risk, they have not eliminated it. Lowering the current (either through LAMP, MST, or iLAST) while still inducing generalized seizures allows us to test the hypothesis that it is the seizure that drives efficacy while the E-field drives side effects. Results to date with MST demonstrating efficacy with minimal cognitive impairment support this hypothesis, that it is the seizure, not the E-field, that is key to efficacy. Non-seizure interventions, such as TMS and TEST, allow us to examine the role of the E-field itself in the absence of seizure. TMS induces weak focal E-fields that are effective for depression and OCD with minimal side effects, though they lack the broad therapeutic spectrum that ECT carries in severe treatment resistant conditions and psychotic subtype. This argues that weak E-fields alone are insufficient to drive efficacy in severe conditions, and further, that the weak E-fields used to induce the seizure in MST likely do not contribute to its efficacy. TEST increases the E-field current amplitude without inducing a seizure to test the opposite hypothesis that it is the E-field that drives efficacy while the seizure drives side effects. Open-label results with TEST are promising, demonstrating efficacy with lower side effects. We await results of the first randomized controlled trial to validate that finding.

So what is our conclusion on the E-field versus seizure debate in ECT? We have presented support for both sides of this argument, and ultimately the answer will be informed by the results of a few consequential trials currently underway. If the confirmatory efficacy trial of MST currently underway shows that MST is as effective as ECT but without the cognitive side effect burden, that would argue strongly for the E-field driving side effects and the seizure driving the efficacy of ECT. If the blinded trial of TEST demonstrates efficacy with lower side effects, then our answer may be more nuanced. Should both TEST and MST carry therapeutic benefit with minimal side effects, we could conclude that strong E-fields and seizures are each sufficient to carry therapeutic benefit on their own, but when given in combination, the interaction between the two results in cognitive side effects.

## Future research directions

Further directions of research may pay closer attention to characterizing the spatial and temporal aspects of the train of E-field pulses as it interacts with the spatial and temporal aspects of the evolving seizure. It is important to appreciate that in the case of ECT (and MST/iLAST, for that matter) the seizure starts during the stimulation train, so in the latter parts of the train E-fields are being applied to a seizing brain. This raises the tantalizing possibility that the interaction between the two could influence the way that the seizure evolves, and/or the susceptibility of the seizing brain to the applied E-field. Better characterizing the spatial/temporal interplay between E-field and seizure may lead to as yet unanticipated discoveries regarding the mechanisms of action of our oldest somatic therapy in psychiatry. New tools will be needed to empirically study this interaction and take advantage of it to optimize therapeutic outcomes. Such research could enhance understanding of how E-fields, and the E-field–seizure interaction, impacts neuronal function at a cellular, sub-cellular, and biophysical level. Unpacking the mechanisms of ECT is key to optimizing the safety of this highly effective intervention, and removing barriers to its use in patients who could benefit.
